# The Practical Medicine Series of Year Books

**Published:** 1907-04

**Authors:** 


**The Practical Medicine Series of Year Books.** Ten volumes. Issued under the general editorial charge of **Gustavus P. Head,** M.D., Professor of Laryngology and Rhinology in the Chicago Post-Graduate Medical School. Vol. IX. Anatomy, Physiology, Pathology, Dictionary. Edited by **W. A. Evans, Adolph Gehrmann,** and **William Healy.** Series 1906. Chicago: The Year Book Publishers. (Price, $1.25; entire series, $10.00).

The new material in the departments of anatomy, physiology, pathology and bacteriology has been gathered together in compact form in this volume and in such manner as to make the presentation of the subjects absolutely complete. The dictionary section contains all the new words which have either crept or been jammed into scientific literature during the year and there has been nothing overlooked, not even the curiosities. The book is an interesting and rather strong link in the chain of year books.

